# Association of fasting blood glucose to high-density lipoprotein cholesterol ratio with short-term outcomes in patients with acute coronary syndrome

**DOI:** 10.1186/s12944-021-01618-2

**Published:** 2022-01-30

**Authors:** Simin Deng, Zhaojun Wang, Yifeng Zhang, Ying Xin, Cheng Zeng, Xinqun Hu

**Affiliations:** Department of Cardiovascular Medicine, The Second Xiangya Hospital, Central South University, 139 Middle Renmin Road, Changsha, 410011 Hunan China

**Keywords:** Major adverse cardiovascular events, Acute coronary syndrome, Fasting blood glucose, High-density lipoprotein cholesterol, Metabolic syndrome, Cardiovascular death

## Abstract

**Background:**

Biochemical markers are crucial for determining risk in coronary artery disease (CAD) patients; however, the relationship between fasting blood glucose to high-density lipoprotein cholesterol (FG/HDL-C) ratio and short-term outcomes in acute coronary syndrome (ACS) patients remains unknown. Therefore, we have investigated the relationship between the FG/HDL-C ratio and short-term outcomes in ACS patients.

**Methods:**

We used data from a pragmatic, stepped-wedge, cluster-randomized clinical trial to perform a post hoc analysis. A total of 11,284 individuals with ACS were subdivided into quartiles according to their FG/HDL-C ratios. We used a multivariate logistic regression model, two-piecewise linear regression model, and generalized additive model (GAM) to evaluate the relationship between the FG/HDL-C ratio and short-term outcomes (major adverse cardiovascular events [MACEs] and cardiovascular [CV] death within 30 days).

**Results:**

The FG/HDL-C ratio was remarkably linked to an enhanced risk of MACEs and CV death in individuals with ACS in the highest quartile (MACEs, odds ratio [OR]: 1.49; 95% confidence interval [CI], [1.11, 1.99]; *P* < 0.01; CV death, OR: 1.69; 95% CI, [1.01, 1.41]; *P* = 0.04). The GAM suggested that the relationship between the FG/HDL-C ratio and MACEs and CV death was non-linear. The two-piecewise linear regression model demonstrated that the threshold values were 3.02 and 3.00 for MACEs and CV death, respectively.

**Conclusions:**

A higher FG/HDL-C ratio is associated with a higher risk of MACEs and CV death in patients with ACS.

**Supplementary Information:**

The online version contains supplementary material available at 10.1186/s12944-021-01618-2.

## Background

Coronary artery disease (CAD) has complex pathophysiological features and is thought to be a multifactorial disease [1, 2]. Globally, CAD contributes to one-third of the deaths in people over the age of 35 years [3]. In particular, acute coronary syndrome (ACS) is considered a serious concern among patients with CAD because of adverse outcomes during the follow-up period, such as death, reinfarction, stroke, major bleeding, and cardiovascular (CV) death [4].

Metabolic syndrome (MetS) is known as the major etiological contributor to CAD [5]. In the pathophysiology of MetS, the widely accepted hypothesis includes abdominal obesity, insulin resistance, hypertension, and dyslipidemia. It specifically manifests as elevated arterial blood pressure, elevated triglycerides (TRIG), elevated fasting blood glucose (FG), and lower high-density lipoprotein cholesterol (HDL-C) [6]. Moreover, each component of the MetS is independently and individually connected to a higher risk of CV events and mortality [7–10].

Biochemical biomarkers are crucial for establishing a diagnosis, determining the risk, and guiding therapy in several clinical scenarios. As a result, a growing number of studies have investigated the association between biochemical markers and adverse consequences in patients with CAD over recent years and several studies reported that elevated FG levels are strongly related to adverse consequences in ACS patients, regardless of the presence of diabetes [11, 12]; similar findings have also been reported for lower HDL-C levels [13]. As a composite indicator, the FG/HDL-C ratio consists of two major indicators: elevated FG levels and lower HDL-C levels attributed to poor prognosis in ACS patients. However, whether the combined effect of the two indicators is greater than that of a single indicator has remained unclear. A recent study from 2020 has proven that the FG/HDL-C ratio is a novel indicator of adverse consequences in non-diabetic CAD patients who have undergone percutaneous coronary intervention (PCI) [14]. However, the FG/HDL-C ratio has not been validated in other populations. To the best of our knowledge, the relationship between the FG/HDL-C ratio and major adverse cardiovascular events (MACEs) and CV death has not been established in ACS patients. Therefore, we analyzed 11,284 individuals with ACS and sought to determine the relationship between the FG/HDL-C ratio and short-term outcomes and discussed the impact of the FG/HDL-C ratio on MACEs and CV death in ACS patients.

## Methods

### Study population

**Fig. 1:**
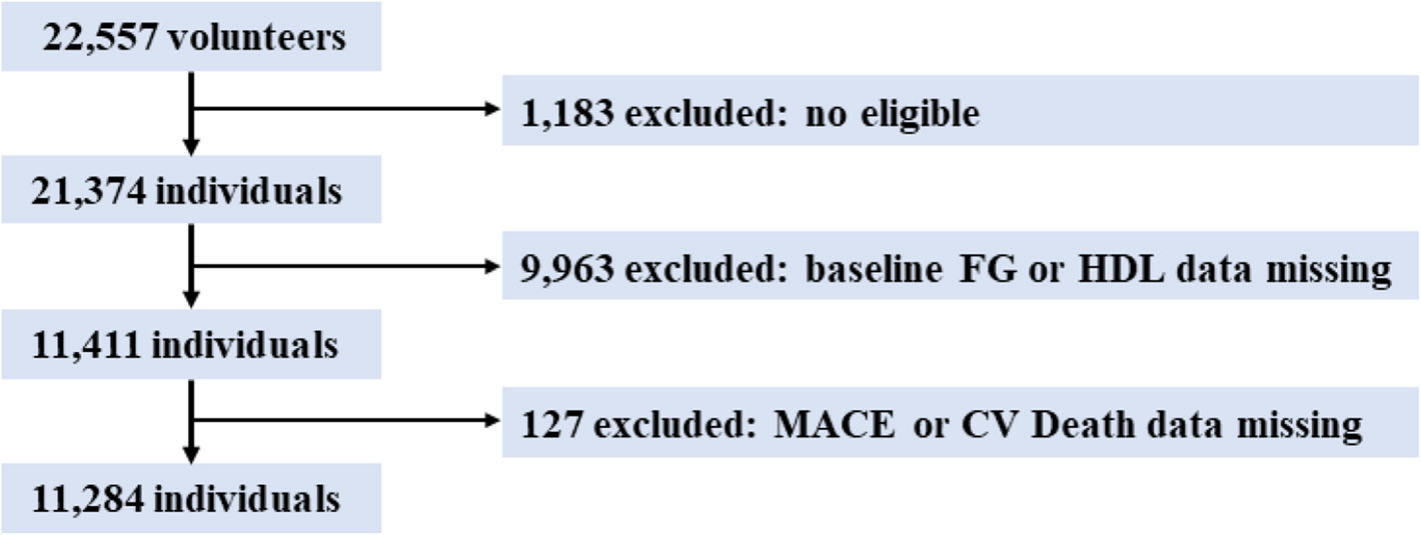
Study population

This was a post hoc analysis of data from a pragmatic, stepped-wedge, and cluster-randomized, clinical trial. The protocol and results of the Acute Coronary Syndrome Quality Improvement in Kerala (ACS-QUIK) study have been described and published previously [15, 16]. In brief, the ACS-QUIK study assessed the application and impact of a locally developed quality improvement toolkit on 30-day MACEs in patients admitted with ACS in Kerala, India. From November 2014 to December 2017, the trial recruited 22,557 volunteers from 63 hospitals; of these, 21,374 were eligible. However, after adjusting for cluster and temporal trends, in comparison to usual care, implementing the quality improvement toolkit did not reduce the 30-day MACEs rates. We obtained a limited dataset from the Biologic Specimen and Data Repository Information Coordinating Center. After excluding 9963 individuals without baseline FG or HDL-C data and 127 individuals without MACEs or CV death data, we analyzed 11,284 individuals in this study (Fig. 1).

### Variables and definitions

The variables we used are shown in Table 1. At the first medical contact, the baseline FG and HDL-C levels were defined as first FG and HDL-C levels. “Intervention” was defined as the use of the quality improvement toolkit. The primary endpoint was 30-day MACEs, defined as death, major bleeding, reinfarction, and stroke. The secondary endpoint was 30-day CV death. “30-day” was defined from the admission to post-discharge 30-days.

**Table 1:** Baseline characteristics of participants

Characteristic	Q1 (***n*** = 2823)	Q2 (***n*** = 2821)	Q3 (***n*** = 2830)	Q4 (***n*** = 2810)	***P***-value
FG/HDL-C ratio	1.88 ± 0.33	2.78 ± 0.25	3.80 ± 0.37	6.52 ± 2.07	< 0.001
Intervention	1222 (43.29%)	1436 (50.90%)	1429 (50.49%)	1405 (50.00%)	< 0.001
Sex					0.006
Female	715 (25.33%)	684 (24.25%)	614 (21.70%)	703 (25.02%)	
Male	2108 (74.67%)	2137 (75.75%)	2216 (78.30%)	2107 (74.98%)	
Age (years)	60.64 ± 12.63	60.40 ± 12.22	59.42 ± 11.81	59.97 ± 11.50	< 0.001
Heart rate (BPM)	78.71 ± 18.21	79.68 ± 19.40	80.10 ± 19.08	82.93 ± 20.46	< 0.001
Weight (kg)	62.31 ± 9.36	63.13 ± 9.71	64.16 ± 9.77	64.22 ± 9.59	< 0.001
SBP (mmHg)	138.49 ± 27.74	141.78 ± 28.71	140.78 ± 29.49	139.85 ± 29.92	< 0.001
Hemoglobin (g/dL)	13.17 ± 1.99	13.36 ± 1.97	13.47 ± 1.99	13.18 ± 2.09	< 0.001
CK-MB (units/L)	65.77 ± 83.07	71.26 ± 91.22	74.03 ± 93.68	64.66 ± 88.11	0.087
Troponin (ng/mL)	8.80 ± 22.86	10.88 ± 24.20	11.55 ± 26.99	9.33 ± 22.56	0.471
sCr (units/L)	1.16 ± 0.54	1.14 ± 0.54	1.17 ± 0.65	1.22 ± 0.74	0.020
HDL-C (mg/dL)	49.53 ± 11.04	42.65 ± 8.48	39.06 ± 8.99	36.09 ± 9.11	< 0.001
LDL-C (mg/dL)	125.08 ± 37.83	125.05 ± 40.55	123.17 ± 40.63	118.47 ± 41.81	< 0.001
TRIG (mg/dL)	130.37 ± 60.91	129.67 ± 64.97	138.14 ± 71.63	153.52 ± 84.79	< 0.001
FG (mg/dL)	91.78 ± 20.83	118.36 ± 24.42	148.15 ± 36.61	230.90 ± 75.54	< 0.001
Smoking or tobacco	1156 (40.95%)	860 (30.49%)	855 (30.21%)	709 (25.23%)	< 0.001
Hypertension	1132 (40.10%)	1249 (44.28%)	1397 (49.36%)	1424 (50.68%)	< 0.001
PAD	18 (0.64%)	31 (1.10%)	26 (0.92%)	44 (1.57%)	0.006
Prior TIA or stroke	78 (2.76%)	66 (2.34%)	53 (1.87%)	79 (2.81%)	0.079
Diabetes	652 (23.10%)	946 (33.53%)	1556 (54.98%)	2242 (79.79%)	< 0.001
STEMI	1751 (62.03%)	1884 (66.78%)	1892 (66.86%)	1841 (65.52%)	< 0.001
Heart failure	181 (6.41%)	261 (9.25%)	289 (10.21%)	309 (11.00%)	< 0.001
Cardiac shock	49 (1.74%)	38 (1.35%)	60 (2.12%)	95 (3.38%)	< 0.001
Cardiac arrest	26 (0.92%)	21 (0.74%)	36 (1.27%)	38 (1.35%)	0.085
Killip class					< 0.001
I	2499 (88.52%)	2456 (87.06%)	2429 (85.83%)	2324 (82.70%)	
II	86 (3.05%)	157 (5.57%)	164 (5.80%)	184 (6.55%)	
III	155 (5.49%)	168 (5.96%)	190 (6.71%)	244 (8.68%)	
IV	83 (2.94%)	40 (1.42%)	47 (1.66%)	58 (2.06%)	
LVEF category					< 0.001
1	281 (9.95%)	363 (12.87%)	408 (14.42%)	505 (17.97%)	
2	2083 (73.79%)	1996 (70.76%)	1971 (69.65%)	1908 (67.90%)	
3	142 (5.03%)	166 (5.88%)	191 (6.75%)	137 (4.88%)	
4	317 (11.23%)	296 (10.49%)	260 (9.19%)	260 (9.25%)	
Symptom onset to arrival (min)	809.61 ± 1263.34	763.32 ± 1269.04	773.15 ± 1272.50	783.14 ± 1292.02	0.573
Door to balloon (min)	538.33 ± 1421.01	483.20 ± 1204.73	510.32 ± 1513.07	511.91 ± 1489.49	0.832
Angiography	1313 (46.51%)	1942 (68.84%)	2067 (73.04%)	1989 (70.78%)	< 0.001
PCI	1000 (35.42%)	1615 (57.25%)	1735 (61.31%)	1640 (58.36%)	< 0.001
CABG	8 (0.28%)	20 (0.71%)	15 (0.53%)	14 (0.50%)	0.162
Aspirin	2350 (97.31%)	2595 (97.34%)	2623 (97.73%)	2529 (97.49%)	0.060
Clopidogrel	2046 (84.72%)	2120 (79.52%)	2072 (77.20%)	2042 (78.72%)	< 0.001
Ticagrelor	223 (9.23%)	347 (13.02%)	378 (14.08%)	344 (13.26%)	< 0.001
Beta blocker	1392 (57.64%)	1600 (60.02%)	1703 (63.45%)	1607 (61.95%)	< 0.001
Warfarin	30 (1.24%)	48 (1.80%)	66 (2.46%)	50 (1.93%)	0.059
ACEI	769 (31.84%)	963 (36.12%)	962 (35.84%)	873 (33.65%)	0.001
ARB	202 (8.36%)	233 (8.74%)	240 (8.94%)	243 (9.37%)	0.018
Statin	2340 (96.89%)	2557 (95.91%)	2583 (96.24%)	2513 (96.88%)	0.222

The definition of variables based on the American College of Cardiology/American Heart Association’s key data elements and definitions for measuring the clinical management and outcomes in patients with ACSs and coronary artery disease. BPM: beats per minute. SBP: systolic blood pressure. CK-MB: creatine kinase isoenzymes in the heart. sCr: serum creatinine. HDL-C: high-density lipoprotein cholesterol. LDL-C: low-density lipoprotein cholesterol. TRIG: triglycerides. FG: fasting glucose. PAD: peripheral arterial disease. TIA: transient ischemic attack. STEMI: ST-segment elevation myocardial infarction. LVEF: left-ventricular ejection fraction. LVEF category: 1: ≤40%; 2: 40–70%; 3: ≥70%; 4 = unknown or not assessed. PCI: percutaneous coronary intervention. CABG: coronary-artery bypass graft surgery. ACEI: angiotensin-converting enzyme inhibitor. ARB: Angiotensin receptor blocker

### Statistical analysis

All statistical analyses were conducted using the statistical software packages R (The R Foundation, Vienna, Austria) and EmpowerStats (X&Y Solutions, Inc., Boston, USA). We used normal quantile-quantile plots to determine whether the continuous variables were normally distributed. Normally distributed variables were presented as means and standard deviations (SDs). Not normally distributed variables were reported as medians with interquartile ranges. Frequencies with percentages were used to describe categorical variables. To determine statistical differences between variables, one-way analysis of variance or the Kruskal-Wallis H test was used for continuous variables and chi-square tests were used for categorical variables.

Multiple logistic regression models were used to evaluate the relationship between the FG/HDL-C ratio and MACEs and CV death, including non-adjusted, minor-adjusted, and fully adjusted models. We carefully selected confounders in accordance with their clinical implications, statistically significant differences in the univariable analysis (Supplement Tables 1 and 2), and their change in effect estimate of more than 10%. To verify if the findings were robust, both non-adjusted and multivariate-adjusted models were used, and the interaction and stratified analyses were performed. Meanwhile, we used the FG/HDL-C ratio as either a continuous or categorical variable to evaluate the relationship, and the first quartile was chosen as the reference when the FG/HDL-C ratio was handled as a categorical variable. The generalized additive model (GAM) was used to evaluate the non-linear relationship between the FG/HDL-C ratio and 30-day endpoints (if the equivalent degrees of freedom were > 1, a nonlinear relationship was determined). The two-piecewise linear regression model was used to calculate the threshold effect of the relationship between the FG/HDL-C ratio and 30-day endpoints. The inflection point was calculated automatically by the recursive method using the maximum-likelihood model. On the inflection point’s left and right sides, there existed different regression coefficients. Finally, we used the logarithmic likelihood ratio test to evaluate whether one-line or two-piecewise linear regression models were more appropriate for describing the data.

## Results

### Baseline characteristics of participants

All individuals were subdivided into four quartiles according to the FG/HDL-C ratios. The baseline characteristics of the study population are shown in Table 1. Briefly, a total of 11,284 individuals (8568 male and 2716 female) were available for the final baseline analysis. Their age was 60.11 ± 12.05 years; 5202 individuals (46.10%) had a history of hypertension, and 5396 individuals (47.82%) had a history of diabetes. The FG/HDL-C ratio was 3.75 ± 2.05. The following variables were statistically significantly different (*P* < 0.05) among the four quartiles: intervention, sex, age, heart rate, weight, SBP, hemoglobin, sCr, HDL-C, LDL-C, TRIG, FG, smoking or tobacco, hypertension, PAD, diabetes, Killip class, LVEF category, STEMI, heart failure, cardiac shock, angiography, PCI, and the use of medications including clopidogrel, ticagrelor, beta-blocker, ACEI, ARB. No statistically significant differences were found between the other indicators. Individuals with FG/HDL-C ratios in the highest quartile had much higher sCr and FG and lower HDL-C and LDL-C. Fewer of them smoked. Meanwhile, they had a higher incidence of hypertension, PAD, diabetes, heart failure, and cardiac shock.

### Relationship between the FG/HDL-C ratio and short-term outcomes

The multivariable stepwise analysis of the three models is shown in Table 2. In individuals in the highest quartile, the FG/HDL-C ratio was strongly linked to an elevated risk of MACEs (non-adjusted model, odds ratio [OR]: 1.42; 95% confidence interval [CI], [1.10, 1.82]; *P* < 0.01) (minor-adjusted model, OR: 1.57; 95% CI, [1.21, 2.03]; *P* < 0.01) (fully adjusted model, OR: 1.49; 95% CI, [1.11, 1.99]; *P* < 0.01). Regarding CV death, individuals in the highest quartile had the highest risk (non-adjusted model, OR: 1.56; 95% CI, [1.16, 2.10]; *P* < 0.01) (minor-adjusted model, OR: 1.77; 95% CI, [1.30, 2.39]; *P* < 0.01) (fully adjusted model, OR: 1.69; 95% CI, [1.01, 1.41); *P* = 0.04) in comparison to those in the lowest quartile. When we used the FG/HDL-C ratio as a continuous covariate, in the non-adjusted model, the risk of MACEs and CV death increased as the FG/HDL-C ratio increased (MACEs, OR: 1.09; 95% CI, [1.05, 1.13]; *P* < 0.01) (CV death, OR: 1.11; 95% CI, [1.07, 1.16); *P* < 0.01). There was no major change in the outcomes after minor adjustment (MACEs, OR: 1.10; 95% CI, [1.06, 1.15]; *P* < 0.01) (CV death, OR: 1.13; 95% CI, [1.08, 1.18); *P* < 0.01). In the fully adjusted model, fully adjusting for confounders also did not change the trend (MACEs, OR: 1.09; 95% CI, [1.04, 1.14]; *P* < 0.01) (CV death, OR: 1.11; 95% CI, [1.04, 1.19); *P* < 0.01).

**Table 2:** Relationship between the FG/HDL-C ratio and short-term outcomes in different models

MACEs			
Exposure	Non-adjusted ^**a**^	Minor-adjusted ^**b**^	Fully adjusted ^**c**^
FG/HDL-C ratio	1.09 (1.05, 1.13), *P* < 0.01	1.10 (1.06, 1.15), *P* < 0.01	1.09 (1.04, 1.14), *P* < 0.01
FG/HDL-C ratio grouping			
Q1	Ref	Ref	Ref
Q2	0.93 (0.70, 1.22), *P* = 0.58	0.95 (0.72, 1.26), *P* = 0.75	0.98 (0.73, 1.30), *P* = 0.88
Q3	0.89 (0.68, 1.18), *P* = 0.43	1.00 (0.76, 1.33), *P* = 0.98	1.03 (0.76, 1.38), *P* = 0.86
Q4	1.42 (1.10, 1.82), *P* < 0.01	1.57 (1.21, 2.03), P < 0.01	1.49 (1.11, 1.99), *P* < 0.01
**CV death**			
**Exposure**	**Non-adjusted** ^**a**^	**Minor-adjusted** ^**b**^	**Fully adjusted** ^**d**^
FG/HDL-C ratio	1.11 (1.07, 1.16), *P* < 0.01	1.13 (1.08, 1.18), *P* < 0.01	1.11 (1.04, 1.19), *P* < 0.01
FG/HDL-C ratio grouping			
Q1	Ref	Ref	Ref
Q2	0.95 (0.68, 1.31), *P* = 0.74	0.98 (0.70, 1.38), *P* = 0.92	1.01 (0.59, 1.71), *P* = 0.98
Q3	0.82 (0.58, 1.15), *P* = 0.26	0.94 (0.66, 1.33), *P* = 0.71	0.83 (0.48, 1.46), *P* = 0.53
Q4	1.56 (1.16, 2.10), *P* < 0.01	1.77 (1.30, 2.39), *P* < 0.01	1.69 (1.01, 1.41), *P* = 0.04

Co-linearity analysis showed that FG, cardiac arrest, PCI, CABG and FG/HDL-C ratio had high co-linearity. Therefore, FG, cardiac arrest, PCI, and CABG weren’t included in multivariate model

^a^No adjustment

^b^Adjusted for cohort, intervention, age, and sex

^c^Adjusted for cohort, intervention, age, sex, heart rate, weight, SBP, hemoglobin, TRIG, smoking or tobacco, hypertension, prior TIA or stroke, diabetes, heart failure, cardiac shock, Killip class, LVEF category

^d^Adjusted for cohort, intervention, age, sex, weight, heart rate, troponin, TRIG, smoking or tobacco, hypertension, diabetes, heart failure, cardiac shock, Killip class, LVEF category, symptom onset to arrival

### Analyses of the non-linear relationship

**Fig. 2:**
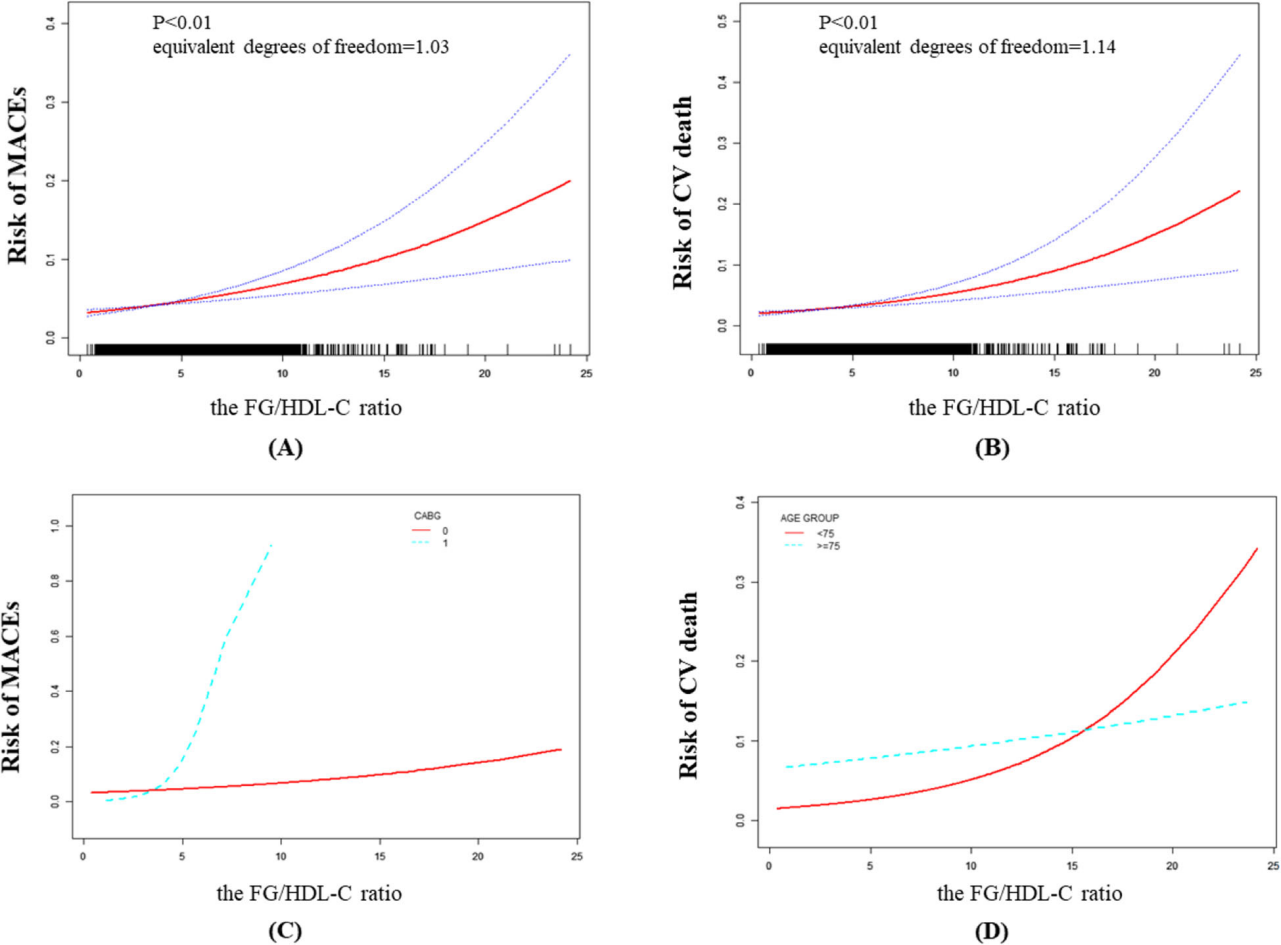
Relationship between the FG/HDL-C ratio and MACEs (**A**), and CV death (**B**); Relationship between the FG/HDL-C ratio and MACEs grouped by CABG (**C**), and CV death grouped by age (**D**). The red line is the trend line, and the blue line is the 95% confidence interval. The denser the vertical lines, the greater the number of patients in the area. CABG 0: without CABG, CABG 1: with CABG

The GAM suggested non-linear relationships between the FG/HDL-C ratio and MACEs and CV death according to the equivalent degrees of freedom, which were 1.03 for MACEs and 1.14 for CV death (Fig. 2 (A) and (B)). We then used two-piecewise linear regression models to characterize the non-linear relationship (Table 3). In addition, we identified 3.02 and 3.00 as the threshold values for MACEs and CV death, respectively. Below these threshold values, the FG/HDL-C ratio was not linked to MACEs or CV death. However, above these threshold values, as the FG/HDL-C ratio increased, and the risk of MACEs and CV death increased (MACEs, OR: 1.12; 95% CI, [1.07, 1.17]; *P* < 0.01; CV death, OR: 1.15; 95% CI, [1.09, 1.20]; *P* < 0.01).

**Table 3:** Results of two-piecewise linear-regression model

(A)	
Outcome: MACEs	
**Exposure: FG/HDL-C ratio**	
One linear-regression model	1.09 (1.05, 1.13), *P* < 0.01
Inflection point (K)	3.02
<K Effect size β (95%CI)	0.88 (0.72, 1.07), *P* = 0.20
>K Effect size β (95%CI)	1.12 (1.07, 1.17), *P* < 0.01
P for Log likelihood ratio test	0.03
(B)	
**Outcome: CV Death**	
**Exposure: FG/HDL-C ratio**	
One linear-regression model	1.11 (1.07, 1.16), *P* < 0.01
Inflection point (K)	3.00
<K Effect size β (95%CI)	0.85 (0.67, 1.08), *P* = 0.20
>K Effect size β (95%CI)	1.15 (1.09, 1.20), *P* < 0.01
P for Log likelihood ratio test	0.03

(A) MACEs: major adverse cardiovascular events and (B) CV death: cardiovascular death. The two-piecewise linear regression model was used to calculate the threshold effect

### Stratified analysis and interaction test

To test the stability of FG/HDL-C ratio among different populations, a stratified analysis and an interaction test were performed. The results are illustrated in Supplementary Figs. 1 and 2. For MACEs, the significant variable in the interaction test was CABG (*P* < 0.01). The OR without CABG was less than that with CABG (1.09 vs. 2.59), and the 95% CIs did not overlap (1.04, 1.13; *P* < 0.01 vs. 1.17, 5.72; *P* = 0.02). For CV death, the significant variable in the interaction test was age (*P* < 0.05). The ORs of patients aged ≥75 years were less than those of patients aged < 75 years (1.04 vs. 1.15); however, the 95% CIs overlapped (1.10, 1.21; *P* < 0.01 vs. 0.95, 1.14; *P* = 0.39). A higher FG/HDL-C ratio indicated a higher risk of MACEs in patients with CABG than those without CABG (Fig. 2 (C)). In patients aged < 75 years, a higher FG/HDL-C ratio indicated a higher risk of CV death than that in those aged ≥75 years (Fig. 2 (D)).

## Discussion

**Fig. 3:**
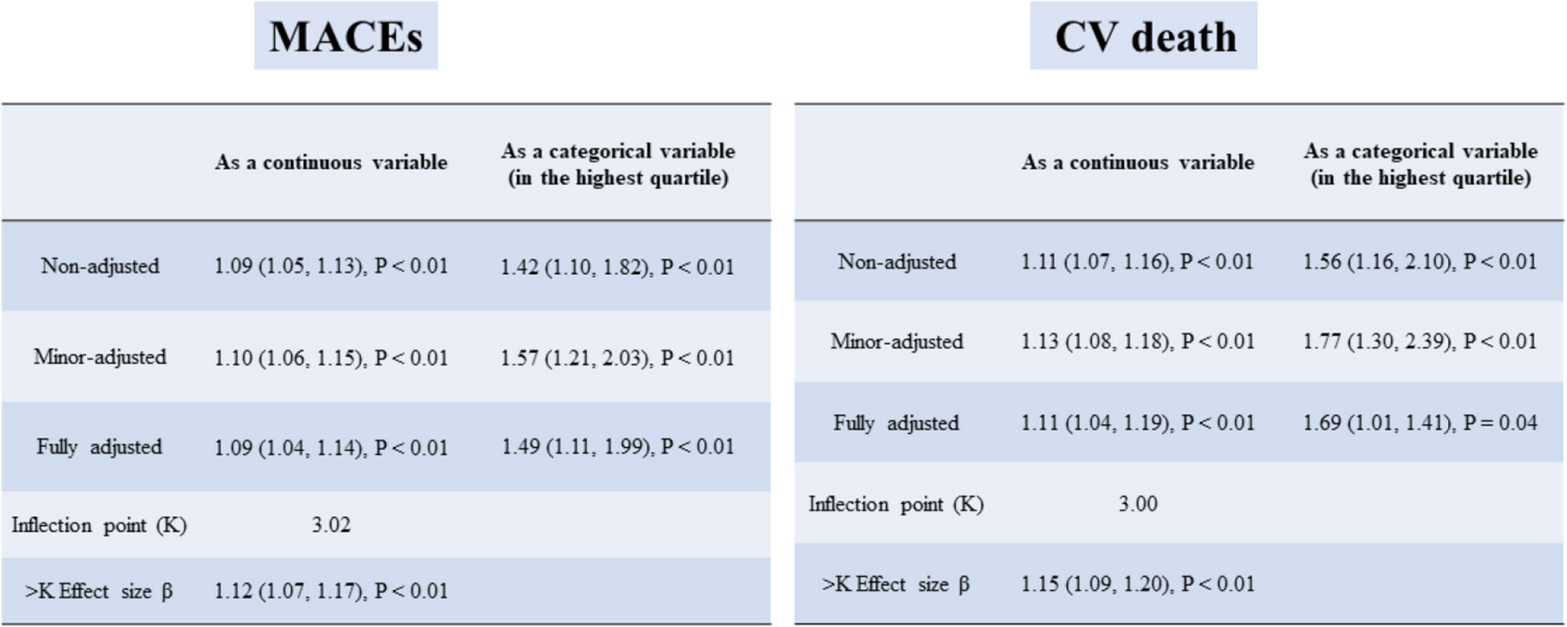
The major findings

We investigated the relationships between the FG/HDL-C ratio and MACEs and CV death in ACS patients. For the first time, our study proven that the incidence of 30-day MACEs and 30-day CV death was higher among individuals with ACS with a FG/HDL-C ratio in the highest quartile. For MACEs, the inflection point was 3.02; when the FG/HDL-C ratio was > 3.02, the incidence of MACEs increased by 12% for every increase of 1 in the FG/HDL-C ratio. For CV death, the inflection point was 3.00; when the FG/HDL-C ratio was > 3.00, the incidence of CV death increased by 15% for every increase of 1 in the FG/HDL-C ratio (Fig. 3). Therefore, an elevated FG/HDL-C ratio was linked to a higher risk of 30-day MACEs and CV death in ACS patients.

Several novel biomarkers have been reported in recent years, including microRNAs [17] and long non-coding ribonucleic acids [18]. However, measuring these biomarkers is expensive, time-consuming, and complicated. Therefore, several limitations should be considered when searching for better biomarkers. In intensive biomarker research studies, some biomarkers showed statistically significant results for adverse consequences in patients with CAD, such as red blood cell distribution width [19], gamma-glutamyl transferase-to-platelet ratio [20], mean platelet volume [21], monocyte-to-HDL ratio [22], triglyceride/HDL-C ratio [23], and triglyceride-glucose index [24]. These biomarkers are more accessible, convenient, and economical and can serve to supplement traditional prognostic indicators and provide new information for clinical assessment and treatment; however, their sensitivity and specificity vary.

During the pathophysiological process leading to CAD, glycoxidation products from hyperglycemia result in superoxide overproduction in arterial endothelial cells leading to inflammation and impaired endothelial function [25]. Meanwhile, when arterial endothelial cells encounter dyslipidemia, the adhesion molecule expression is augmented, and the adhesion of blood leukocytes to the inner surface of the arterial wall is promoted [26]. Elevated FG levels and lower HDL-C levels are known risk factors that are strongly linked to adverse consequences in ACS patients. Farhan et al. reported that among patients with high-risk non-STEMI ACS, for every 1 mg/dL increase in the FG, the hazard ratio (HR) for 1-year death increased by 53% [27]. Similar findings were reported by other investigators; Ramos et al. demonstrated that FG is associated with MACEs in older and younger patient groups (< 65 years and ≥ 65 years) [28]. However, a relatively small number of patients were included, only 580 patients. Statins are widely prescribed for the treatment of CVD. Statin treatment slightly increases serum HDL-C levels; however, there is a paradoxical decrease in HDL-C levels in some clinical settings. In a previous study, the HR for MACEs increased by 56% for every 1 mg/dL reduction in HDL-C in patients initiated on statin treatment [29]. Similarly, Hirayama et al. demonstrated that compared with LDL-C levels, HDL-C levels were more relevant in the incidence of MACEs, especially in patients with stable angina pectoris [30]. The follow-up in the trial by Hirayama et al. lasted for up to 7 years. Overall, these findings extend the previously reported findings from other investigators.

Recently, the relation between the FG/HDL-C ratio and clinical adverse consequences was studied in patients without diabetes after PCI [14]. The findings suggested that the incidence of all-cause mortality increased by 28.4% in individuals with a higher FG/HDL-C ratio. However, few studies have been conducted on the FG/HDL-C ratio in other populations. Therefore, our study focused on ACS patients regardless of whether they had diabetes. Our multivariate logistic regression analysis demonstrated that an increased FG/HDL-C ratio was linked to MACEs and CV death, which agree with the findings of Guo et al. [14]. However, it is worth mentioning that further stratified analyses in our study suggested that the ORs of individuals with diabetes were less than those of individuals without diabetes for MACEs, and the ORs of individuals with diabetes were higher than those of individuals without diabetes for CV death (MACEs, 1.08 vs. 1.09; CV death, 1.11 vs. 1.10), although the interaction did not reach statistical significance.

Overall, we analyzed more than 10,000 individuals with ACS in India. According to their FG/HDL-C ratios, all study subjects were classified into four groups. In the baseline characteristic analysis, several characteristics of the four groups were remarkably different, and there may have been some unmeasured differences between the four groups. Considering the effect of these confounders, we carefully conducted multivariate logistic regression analysis to reduce this effect; however, the ORs were not substantially altered. Stratified analysis of our data showed that the increased risk of MACEs was more pronounced in individuals undergoing CABG, and the increased risk of CV death was more pronounced in individuals aged < 75 years. Based on these findings, our study highlights some aspects of the management of ACS patients.

### Comparisons with other studies and what does the current work add to the existing knowledge

Based on the existing knowledge, our findings firstly provided validation of FG/HDL-C ratio in ACS patients, found the non-linear relationship between the FG/HDL-C and short-term outcomes, and identified the inflection point of the non-linear relationship.

### Study strengths and limitations

The number of participants is one major strength of our study; we analyzed 11,284 individuals. The current study is the first report to correlate the FG/HDL-C ratio with MACEs and CV death in ACS patients. Meanwhile, our study had several limitations. First, to minimize the effect of confounders, we adjusted for many variables as much as possible; however, because of the incompleteness of the original data, we cannot ensure that all confounding variables were fully adjusted for. Second, we used data from hospitals in Kerala, India, and many individuals were excluded due to missing data. The external validity of our results is limited. However, a large contemporary trial [31] and a real-world registry [32], including other ethnicities, have shown a similar prevalence of dyslipidemia and other risk factors in the study population. These relevant findings may potentially support the generalizability of our conclusions. Third, the researchers did not use the TIMI score and SYNTAX score to identify the ACS patients in the ACS-QUIK study; this oversight may have led to a conclusion bias in this study. Finally, the follow-up duration in the ACS-QUIK study was only 30 days. It is possible that a longer follow-up may change our conclusions.

## Conclusions

In conclusion, our study demonstrated that the FG/HDL-C ratio may be an accessible, convenient, and economical biomarker of short-term outcomes in ACS patients. In their clinical management, we should pay close attention to ACS patients undergoing CABG and those aged < 75 years with a high FG/HDL-C ratio.

## Supplementary Information

**Additional file 1.** Stratified analysis of MACEs.

## Data Availability

Data are available from the Biologic Specimen and Data Repository Information Coordinating Center (BioLINCC).

## Abbreviations

CAD: coronary artery disease
FG: fasting blood glucose
HDL-C: high-density lipoprotein cholesterol
ACS: acute coronary syndrome
GAM: generalized additive model
MACEs: major adverse cardiovascular events
CV: cardiovascular
MetS: metabolic syndrome
TRIG: triglycerides
PCI: percutaneous coronary intervention
ACS-QUIK: acute coronary syndrome quality improvement in Kerala
BPM: beats per minute
SBP: systolic blood pressure
CK-MB: creatine kinase isoenzymes in the heart
sCr: serum creatinine
LDL-C: low-density lipoprotein cholesterol
PAD: peripheral arterial disease
TIA: transient ischemic attack
STEMI: ST-segment elevation myocardial infarction
LVEF: left ventricular ejection fraction
CABG: coronary artery bypass graft surgery
ACEI: angiotensin-converting enzyme inhibitor
ARB: angiotensin receptor blocker
SDs: standard deviations
