# Supplementary material for: Association of fasting blood glucose to high-density lipoprotein cholesterol ratio with short-term outcomes in patients with acute coronary syndrome
Source: Lipids Health Dis. 2022 Jan 30;21:17. doi: 10.1186/s12944-021-01618-2 (PMC8802470; doi:10.1186/s12944-021-01618-2)
Supplement: Supplementary file 1 — Additional file 1. Stratified analysis of MACEs. [file 12944_2021_1618_MOESM1_ESM.docx]

**Supplement Figure 1.** Stratified analysis of MACEs.


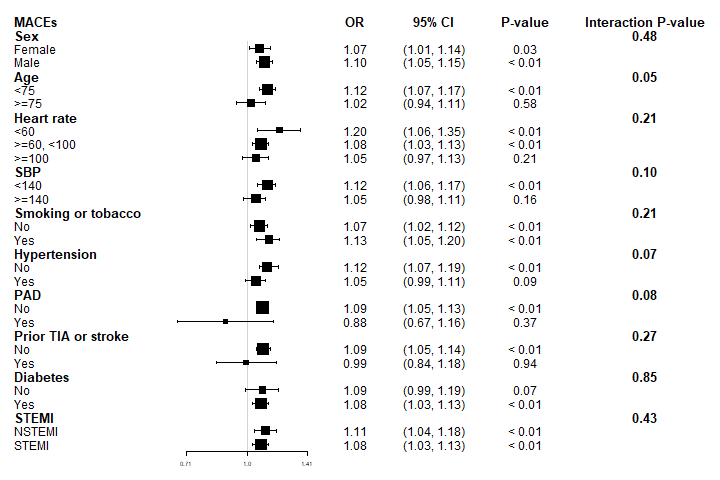


**Continued**


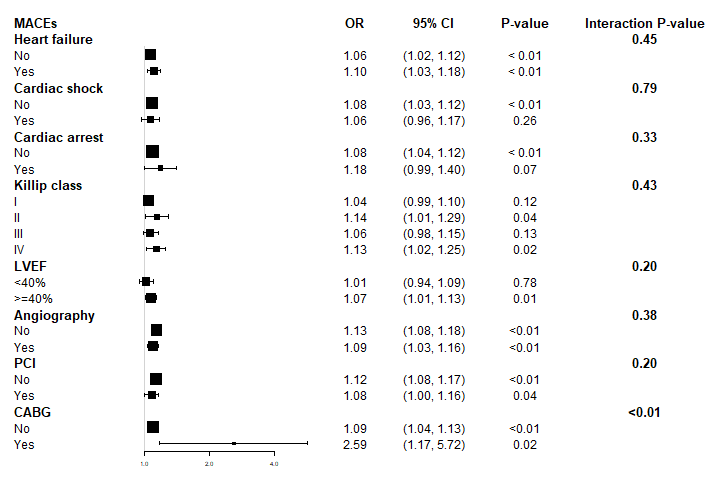


**Supplement Figure 2.** Stratified analysis of CV death.


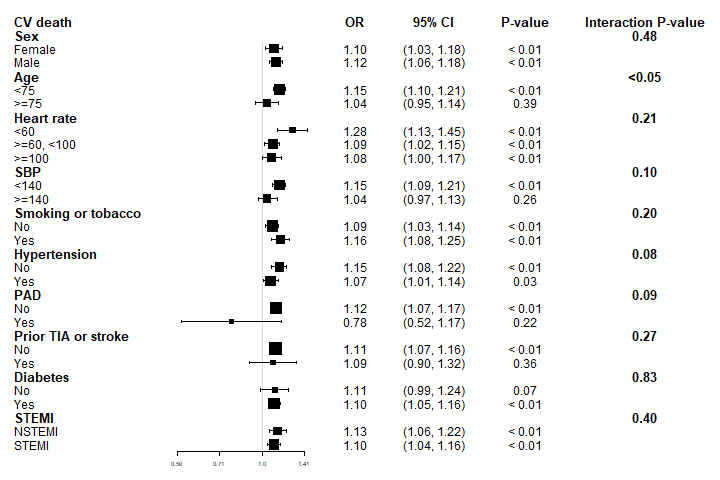


**Continued**


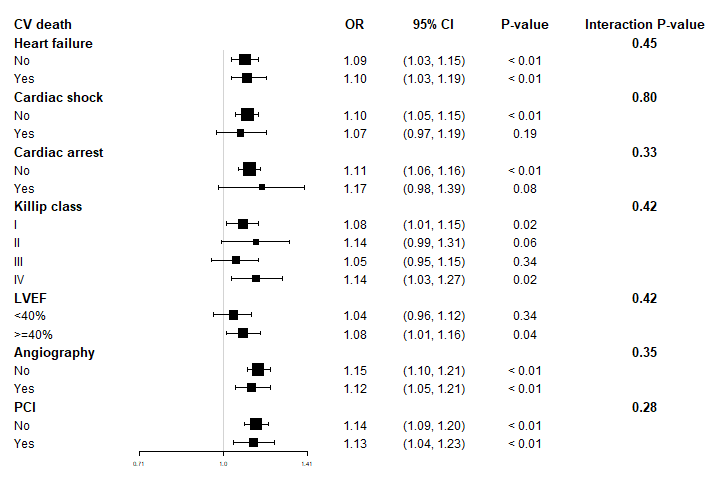


**Supplement Table 1.** Univariate analysis for predicting MACEs

| Characteristic | OR | 95% CI | P-value |
| --- | --- | --- | --- |
| FG/HDL-C index | 1.09 | 1.05-1.13 | <0.001 |
| FG/HDL-C index grouping |  |  |  |
| Q1 | 1.0 |  |  |
| Q2 | 0.93 | 0.70-1.22 | 0.581 |
| Q3 | 0.89 | 0.68-1.18 | 0.430 |
| Q4 | 1.42 | 1.10-1.82 | 0.006 |
| Intervention | 0.88 | 0.73-1.06 | 0.163 |
| Male | 0.44 | 0.36-0.53 | <0.001 |
| Age (years) | 1.05 | 1.04-1.06 | <0.001 |
| Heart rate (BPM) | 1.01 | 1.01-1.0 | <0.001 |
| Weight (kg) | 0.97 | 0.96-0.98 | <0.001 |
| SBP (mmHg) | 0.99 | 0.99-1.00 | 0.001 |
| Hemoglobin (g/dL) | 0.79 | 0.76-0.82 | <0.001 |
| CK-MB (units/L) | 1.00 ( | 1.00-1.00 | 0.116 |
| Troponin (ng/mL) | 1.00 | 1.00-1.01 | 0.046 |
| sCr (units/L) | 1.37 | 1.22-1.53 | <0.001 |
| HDL-C (mg/dL) | 1.01 | 1.00-1.02 | 0.034 |
| LDL-C (mg/dL) | 1.00 | 1.00-1.00 | 0.502 |
| TRIG (mg/dL) | 1.00 | 1.00-1.00 | <0.0001 |
| FG (mg/dL) | 1.00 | 1.00-1.00 | <0.001 |
| Smoking or tobacco | 0.76 | 0.62-0.94 | 0.010 |
| Hypertension | 1.50 | 1.24-1.81 | <0.001 |
| PAD | 3.15 | 1.79-5.54 | <0.001 |
| Prior TIA or stroke | 3.80 | 2.65-5.44 | <0.001 |
| Diabetes | 1.25 | 1.04-1.51 | 0.017 |
| STEMI | 0.91 | 0.75-1.10 | 0.342 |
| Heart failure | 3.10 | 2.47-3.89 | <0.001 |
| Cardiac shock | 6.80 | 4.92-9.41 | <0.001 |
| Cardiac arrest | 10.97 | 7.36-16.34 | <0.001 |
| Killip class |  |  |  |
| I | 1.0 |  |  |
| II | 2.62 | 1.89-3.63 | <0.001 |
| III | 3.92 | 3.03-5.06 | <0.001 |
| IV | 7.60 | 5.37-10.78 | <0.001 |
| LVEF category |  |  |  |
| 1 | 1.0 |  |  |
| 2 | 0.30 | 0.24-0.37 | <0.001 |
| 3 | 0.27 | 0.16-0.44 | <0.001 |
| 4 | 0.61 | 0.45-0.82 | 0.001 |
| Symptom onset to arrival (min) | 1.00 | 1.00-1.00 | 0.007 |
| Door to balloon (min) | 1.00 | 1.00-1.00 | 0.050 |
| Angiography | 0.39 | 0.32-0.47 | <0.001 |
| PCI | 0.37 | 0.30-0.45 | <0.001 |
| CABG | 1.29 | 0.40, 4.13 | 0.673 |
| Aspirin | 0.34 | 0.20-0.59 | <0.001 |
| Clopidogrel | 0.90 | 0.66-1.21 | 0.479 |
| Ticagrelor | 1.20 | 0.84-1.71 | 0.314 |
| Beta Blocker | 0.75 | 0.58-0.97 | 0.026 |
| Warfarin | 4.09 | 2.44-6.84 | <0.001 |
| ACEI | 1.08 | 0.83-1.40 | 0.564 |
| ARB | 1.45 | 0.99-2.12 | 0.058 |
| Statin | 0.76 | 0.41-1.41 | 0.390 |

The definition of variables based on the American College of Cardiology/American Heart Association's key data elements and definitions for measuring the clinical management and outcomes in patients with ACSs and coronary artery disease. BPM: beats per minute. SBP: systolic blood pressure. CK-MB: creatine kinase isoenzymes in the heart. sCr: serum creatinine. HDL-C: high-density lipoprotein cholesterol. LDL-C: low-density lipoprotein cholesterol. TRIG: triglycerides. FG: fasting glucose. PAD: peripheral arterial disease. TIA: transient ischemic attack. STEMI: ST-segment elevation myocardial infarction. LVEF: left-ventricular ejection fraction. LVEF category: 1: ≤40%; 2: 40–70%; 3: ≥70%; 4 = unknown or not assessed. PCI: percutaneous coronary intervention. CABG: coronary-artery bypass graft surgery. ACEI: angiotensin-converting enzyme inhibitor. ARB: Angiotensin receptor blocker.

**Supplement Table 2.** Univariate analysis for predicting CV death.

| Characteristic | OR | 95% CI | P value |
| --- | --- | --- | --- |
| FG/HDL index | 1.11 | 1.07-1.16 | <0.0001 |
| FG/HDL index grouping |  |  |  |
| Q1 | 1.0 |  |  |
| Q2 | 0.95 | 0.68-1.31 | 0.741 |
| Q3 | 0.82 | 0.58-1.15 | 0.255 |
| Q4 | 1.56 | 1.16-2.10 | 0.003 |
| Intervention | 0.78 | 0.62-0.98 | 0.030 |
| Male | 0.38 | 0.30-0.48 | <0.001 |
| Age (years) | 1.06 | 1.05-1.07 | <0.001 |
| Heart Rate (BPM) | 1.02 | 1.01-1.02 | <0.001 |
| Weight (kg) | 0.96 | 0.95-0.97 | <0.001 |
| SBP (mmHg) | 0.99 | 0.99-0.99 | <0.001 |
| Hemoglobin (g/dL) | 0.76 | 0.73-0.80 | <0.001 |
| CK-MB (units/L) | 1.00 | 1.00-1.00 | 0.160 |
| Troponin (ng/mL) | 1.01 | 1.00-1.01 | 0.010 |
| sCr (units/L) | 1.47 | 1.30-1.65 | <0.001 |
| HDL-C (mg/dL) | 1.01 | 1.00-1.02 | 0.203 |
| LDL-C (mg/dL) | 1.00 | 1.00-1.00 | 0.236 |
| TRIG (mg/dL) | 1.00 | 1.00-1.00 | 0.004 |
| FG (mg/dL) | 1.00 | 1.00-1.00 | <0.001 |
| Smoking Or Tobacco | 0.71 | 0.55-0.92 | 0.010 |
| Hypertension | 1.40 | 1.12-1.74 | 0.003 |
| PAD | 2.83 | 1.42-5.63 | 0.003 |
| Prior TIA or Stroke | 3.41 | 2.21-5.27 | <0.001 |
| Diabetes | 1.33 | 1.07-1.67 | 0.011 |
| STEMI | 0.86 | 0.68-1.08 | 0.196 |
| Heart Failure | 3.74 | 2.90-4.84 | <0.001 |
| Cardiac Shock | 8.57 | 6.06-12.14 | <0.001 |
| Cardiac Arrest | 10.55 | 6.77-16.43 | <0.001 |
| Killip Class |  |  |  |
| I | 1.0 |  | 1.0 |
| II | 3.12 | 2.15-4.55 | <0.001 |
| III | 4.73 | 3.52-6.35 | <0.001 |
| IV | 10.56 | 7.27-15.35 | <0.001 |
| LVEF Category |  |  |  |
| 1 | 1.0 |  |  |
| 2 | 0.26 | 0.20-0.34 | <0.001 |
| 3 | 0.24 | 0.13-0.45 | <0.001 |
| 4 | 0.69 | 0.49-0.97 | 0.031 |
| Symptom onset to arrival (min) | 1.00 | 1.00-1.00 | 0.001 |
| Door to balloon (min) | 1.00 | 1.00-1.00 | 0.535 |
| Angiography | 0.28 | 0.22-0.35 | <0.001 |
| PCI | 0.25 | 0.19-0.33 | <0.001 |
| CABG | 0.00 | 0.00-inf. | 0.967 |
| Aspirin | 0.22 | 0.12-0.41 | <0.001 |
| Clopidogrel | 1.01 | 0.65-1.59 | 0.948 |
| Ticagrelor | 1.00 | 0.58-1.73 | 0.998 |
| Beta Blocker | 0.44 | 0.31-0.64 | <0.001 |
| Warfarin | 4.49 | 2.24-9.01 | <0.001 |
| ACEI | 0.96 | 0.65-1.41 | 0.835 |
| ARB | 0.83 | 0.42-1.65 | 0.604 |
| Statin | 0.38 | 0.20-0.74 | 0.004 |

The definition of variables based on the American College of Cardiology/American Heart Association's key data elements and definitions for measuring the clinical management and outcomes in patients with ACSs and coronary artery disease. BPM: beats per minute. SBP: systolic blood pressure. CK-MB: creatine kinase isoenzymes in the heart. sCr: serum creatinine. HDL-C: high-density lipoprotein cholesterol. LDL-C: low-density lipoprotein cholesterol. TRIG: triglycerides. FG: fasting glucose. PAD: peripheral arterial disease. TIA: transient ischemic attack. STEMI: ST-segment elevation myocardial infarction. LVEF: left-ventricular ejection fraction. LVEF category: 1: ≤40%; 2: 40–70%; 3: ≥70%; 4 = unknown or not assessed. PCI: percutaneous coronary intervention. CABG: coronary-artery bypass graft surgery. ACEI: angiotensin-converting enzyme inhibitor. ARB: Angiotensin receptor blocker.
